# Empowering Sedentary Adults to Reduce Sedentary Behavior and Increase Physical Activity Levels and Energy Expenditure: A Pilot Study

**DOI:** 10.3390/ijerph120100414

**Published:** 2015-01-05

**Authors:** Faisal A. Barwais, Thomas F. Cuddihy

**Affiliations:** 1 Department of Physical Education and Sports, Umm Al-Qura University, Makkah 21955, Saudi Arabia; 2 School of Exercise and Nutrition Sciences, Institute of Health and Biomedical Innovation, Queensland University of Technology, Brisbane 4059, Australia

**Keywords:** objective measurement of sedentary behavior, accelerometer, 7-day SLIPA log, self-report physical activity

## Abstract

*Objective*: The purpose of this study was to assess the effectiveness of a 4-week intervention in which an online personal activity monitor (Gruve-Technologies^™^) was used to reduce sedentary behavior among sedentary adults. *Method*: Eighteen, sedentary adult volunteers (12 men, six women, mean age 29 ± 4.0 years) were recruited to participate in the study. Time spent in sedentary activities and light-, moderate-, and vigorous-intensity physical activity and energy expenditure were assessed during waking hours using the monitor and the 7-day SLIPA Log at both baseline and post-intervention. *Results*: A significant decrease of 33% (3.1 h/day; *p* < 0.001) was found between the time spent in sedentary activities measured at baseline (9.4 ± 1.1 h/day) and at the end of the 4-week intervention (6.3 ± 0.8 h/day). Consequent to the changes in sedentary time, significant increases were found in the amount of time spent in light- (45% (2.6 h/day), *p* < 0.001), moderate- (33% (1 h/day)* p* < 0.001), vigorous-intensity physical activity (39% (0.16 h/day), *p* < 0.001), and energy expenditure (47% (216.7 kcal/day), *p* < 0.001). *Conclusion*: This monitor contributes to a meaningful reduction in time spent in sedentary activities and has a large effect on energy expenditure and physical activity patterns.

## 1. Introduction

Due to concerns about declining levels of physical activity and the increasing amount of time people spend in sedentary behavior [1,2], the body of literature addressing the physical activity levels and sedentary behavior of the general population is rapidly growing [3,4]. These negative changes in lifestyle may be due to increases in sedentary lifestyles in the home, at the office, and during leisure time [1]. Such increases in sedentary behavior could also be related to the increasing popularity and availability of new technologies such as entertainment systems and computers [5]. Sedentary behavior has most recently been defined as any waking behavior characterized by an energy expenditure ≤1.5 Metabolic Equivalent Units (METs) while in a sitting or reclining posture [6]. A recent study using an objective measure showed that adults are increasingly sedentary, spending approximately 50%–60% of their day engaged in sedentary activities [7]. Evidence suggests that a dose-response association exists between sitting time and mortality from all causes and cardiovascular disease, independent of leisure time physical activity [8]. In addition, sedentary behaviors are associated with health risks, including various physiological and psychological problems, which are often independent of the time spent on moderate to vigorous activity [9]. Researchers have suggested that sedentary behavior may be a more important indicator of poor health than moderate to vigorous physical activity [10]. In contrast, a growing body of research suggests that breaking up time spent in sedentary activities with short bouts of light- or moderate-intensity walking, or even substituting minimal activities such as sitting with standing, has a positive effect on health, energy and the sense of well-being [11,12,13,14,15].

As a consequence of such research, increased attention is being paid to developing potential intervention methods focused on replacing time spent in sedentary activities with standing and other light-intensity activities of daily living [16,17,18,19,20]. In recent years, technology has come to play a very important role in promoting and supporting an active lifestyle through self-monitoring, goal setting, and other behavioral strategies [21,22]. Recent technological advances have enabled effective motivational applications both for monitoring time spent in sedentary activities and physical activity levels. These applications typically use small, wearable sensors. The data from the sensors can easily be uploaded via an interactive, online interface. Such actions provide users with easy-to-understand visualizations of their daily activity patterns.

The aims of this study were to (1) examine the use of the Gruve online personal activity monitor over a 4-week intervention to reduce sedentary behavior and increase physical activity levels and energy expenditure among sedentary adults during free-living activities, and (2) use a self-report method to examine changes in time spent in sedentary activities and light-intensity physical activity across different life domains (work, transportation, household activities and leisure time).

## 2. Methods

### 2.1. Participants

All participants completed a demographic questionnaire at baseline. Twenty-four adult volunteers (16 men, 8 women, aged 20–36 years) were recruited to participate in the study through advertisements in local newsletters, flyers and emails at a metropolitan university in Brisbane, Australia in October 2012. Pre-screening using the International Physical Activity Questionnaire (IPAQ) self-report questionnaire was used to determine participant eligibility in relation to their volume of sedentary behavior. Only those who reported a high total sitting time, defined as >7 h/day, were invited to participate in the study [23,24]. Written informed consent was obtained and the study was approved by the Human Research Ethics Committee of Queensland University of Technology (Ethics Approval Number: 1200000226). Participation was completely voluntary and participants were informed that they could withdraw at any time during the intervention. Before the start of the intervention, four of the volunteers were excluded from the study sample because they reported a low total sitting time (<7 h/day). Low sitting time was an exclusion criteria. During the intervention, two participants did not comply with the Gruve monitor recharging-battery protocol, thus periods of waking daily life were missing, and their data were removed from analyses after the intervention. As a result, analyses were based on a convenience sample of 18 adults (12 men, 6 women, mean age 29 ± 4.0 years).

### 2.2. Study Design

The data for this research emanated from a larger study which was a randomized, lifestyle intervention for adults. That intervention was designed to reduce sedentary behavior and increase physical activity levels in daily living for sedentary adults and to determine if these changes would also be associated with improvement in total wellness [15]. This research paper focuses only on the Gruve monitor outcomes in the intervention and a pre-experimental, one-group, pretest-posttest design was used. Bonferroni corrections were applied to control the familywise error rate and the difference scores were visually inspected for normality via the relevant histograms. The duration of this study was 5 weeks, which included 1 week of baseline data collection and 4 weeks of intervention. The independent variable was the intervention, and the dependent variable was the 7-day average of time spent in sedentary activities, light-, moderate-, and vigorous-intensity physical activity, and energy expenditure during waking hours.

### 2.3. Description of the Online Personal Activity Monitor

MUVE, Inc. (Anoka, MN, USA) developed the Gruve online personal activity monitor in cooperation with the Mayo Clinic (Rochester, MN, USA) [25]. The monitor is a tri-axial accelerometer system that tracks time spent in daily sedentary and light-, moderate-, and vigorous-intensity physical activity and energy expenditure via a wearable device and an accompanying online service. The small activity monitoring device is similar in size and shape to a pedometer and is designed to be worn on the waistband. It monitors a participant’s daily physical activity at 20 Hz and stores the minute data on the monitor for uploading later through a Universal Serial Bus (USB) port to the interactive online software. These data subsequently provide the participants with an easy-to-understand visualization of daily activity patterns. Goal-setting features are used alongside simple graphs and charts to enhance self-monitoring of energy expenditure. An indicator (a halo bar) on the top of the monitor also highlights the participant’s progress toward their daily goal. The indicator bar provides a Light-Emitting Diode (LED) color corresponding to the participant’s progress towards their daily activity goal. For example, at the beginning of the day, the light bar is red but, as the day progresses, if the participant has been sufficiently active, the color changes from red to yellow to orange to blue and finally to green. The green light indicates that the daily activity goal has been achieved. The proprietary monitoring algorithm employs the participant’s gender, height, weight and age to calculate daily and weekly energy expenditure goals. The goal represented by a green light (known as the “Green Goal”) is a function both of a participant’s resting metabolic rate (RMR) and physical activity level. The online monitor automatically sets the Green Goal based on a participant’s current activity patterns and the number of calories the participant needs to burn every day above the RMR. The Green Goal gradually increases as the physical activity level does. The system analyzes each participant’s progress every day. If the participant has reached the Green Goal more than eight times in the previous 14 days, the goal automatically increases by approximately 20% of total average daily energy expenditure; otherwise, the goal remains the same. An additional feature is the built-in vibrating function that provides a short vibrating pulse to the participant when the monitor senses an extended sitting period. For example, if the participant is sedentary for a lengthy period, the monitor will vibrate to notify them that they have been sedentary and are reaching their Energy Conservation Point (ECP). The ECP marks the point at which the body goes into a reduced caloric burn rate following a prolonged period of sedentary behavior. This point varies from person to person based on the individual’s biometrics [26]. Research has shown that the Gruve personal activity monitor is accurate when measuring energy expenditure, sedentary behavior and walking at seven different velocities in the laboratory [27,28]. Further, Dutta *et al*. [17] found that the Gruve monitor was highly correlated (r^2^ > 0.95) with the Modular Signal Recorder accelerometer (Modular Signal Recorder 145, MSR Electronics GmbH, Seuzach, Switzerland) for the measurement of both sitting and standing. They then suggested that the Gruve would be appropriate for use during free-living conditions.

### 2.4. Intervention Structure

Participants completed a 4-week intervention using the Gruve monitor to reduce sedentary behavior during free-living activities. The intervention simply consisted of the participants regularly logging onto the Gruve online personal activity monitor software and tracking their physical activity level during those 4 weeks. Each participant completed two visits to the university. During the first visit, participants’ height (cm) and weight (kg) were measured. The International Society for the Advancement of Kinanthropometry (ISAK) protocols were followed [29]. Body mass index (BMI) scores were calculated based on weight (in kg) divided by height (in meters2). Participants received detailed instructions for placing the monitor, wearing it, and installing online software on their own computers [25]. Subsequently, participants were asked to create login IDs and add their gender, height, weight and age information to the website. At baseline (assessment week 1), participants wore the monitor for 7 days (5 weekdays and 2 weekend days) during free-living activities, except when sleeping or bathing. Participants were asked to ensure they followed normal daily physical activities and sedentary routines. Additionally, participants were advised to charge the monitor battery at night while sleeping. During this baseline assessment week, the monitor’s halo light bars were constantly green, as were the graphic online bar charts. After baseline, based on the physical activity level determined during the 7-day baseline assessment period, the online personal activity monitor software automatically set Green Goals for the participants and started displaying different colors on the monitor and online charts. Throughout the intervention period (weeks 1 to 4), participants continued to wear the monitor daily both on weekdays and weekend days during free-living activities, except when sleeping, bathing or swimming. To increase their motivation, participants were encouraged to achieve their daily monitor goals (green bar) and view their daily online homepages. Weekly motivational emails from the online system were sent to participants when they achieved their Green Goals, to encourage the participants to continue to be more active. During participants’ second visit to the university (after completion of the 4-week intervention), they returned the monitor.

### 2.5. Study Measures

#### 2.5.1. Objective Measurement

Time spent in sedentary activities and light-, moderate-, and vigorous-intensity physical activity and energy expenditure were measured during waking hours. The Gruve software employs commonly utilized cutoffs as follows: sedentary activity is defined as 0 to 1.5 metabolic equivalent of task (MET), light activity as 1.6 to 3 MET, moderate as 3.1 to 6 MET (e.g., brisk walking), and intense as 6+ MET (e.g., jogging) [25]. Every 3 days, when the participants synced their monitor with online software, the recorded data were downloaded by the first author through logging into the participant’s page on the online software. Data entry was performed in Excel by an independent research assistant and double entered by the first author. The average of the baseline week as well as the average of the last week of the 4-week intervention were recorded and, subsequently, used for analyses.

#### 2.5.2. Self-Report Measurement

Daily sedentary activities and light-intensity physical activity within specific behavioral life domains were measured using the 7-day Sedentary and Light Intensity Physical Activity Log (7-day SLIPA Log) [30]. The 7-day SLIPA Log is a 23-item instrument that collects information about sedentary behavior and light-intensity physical activity across four daily life behavioral domains: domains: work (seven questions), transport (three questions), home (six questions) leisure (six questions) and sleeping (one question). Each question corresponds to a specific level of MET-intensity, according to the Compendium of Physical Activity [31]. One MET (metabolic equivalent) is defined as the energy cost of resting quietly, or often defined in terms of oxygen uptake as 3.5 mL∙kg^‑1^∙min^-1^ [3]. Questions included in the 7-day SLIPA Log were organized into eight different MET levels (0.9–2.5), ranging from 0.9 METs (sleeping) to 2.5 METs (walking).The level of detail the log provides about specific behavioral domains allows researchers to identify where changes in particular behaviors such as sitting or walking may have occurred (e.g., at home or work). In order to avoid/reduce measurement error, participants received daily reminder emails reminding them to complete the 7-day SLIPA Log. Participants were asked to complete the 7-day SLIPA Log by indicating how many hours and minutes they had spent in sedentary behavior and light-intensity physical activity in each of the four daily life behavioral domains for the previous day (12:00 a.m. to 11:59 p.m.). The validity of the 7-day SLIPA Log has been tested against an ActiGraph GT3X accelerometer for 7 consecutive days, and the correlation for time spent in sedentary activities (*r* = 0.86, *p* < 0.001) and for light-intensity physical activity (*r* = 0.80, *p* < 0.001) was acceptable across the four daily life domains [30].

### 2.6. Statistical Analysis

All statistical analyses were carried out using SPSS statistical software version 21.0 for Windows (SPSS Inc., Chicago, IL, USA). Descriptive data were expressed as means and SDs. To determine whether pre-post changes across the intervention differed from baseline to the end of the 4-week intervention, a paired t-test was used. Differences between the average of the amount of time spent in sedentary activities, light-, moderate-, and vigorous-intensity physical activity, and energy expenditure were assessed at baseline and at the end of the 4-week intervention and standardized for wear time using the residuals method [32]. Paired t-tests also were used to determine differences between baseline and the end of the 4-week intervention for the 7-day SLIPA Log across four daily life domains (work, transportation, household activities and leisure time). Effect sizes for paired *t*-tests were calculated following Cohen’s d (small effect ≥ 0.20; medium effect ≥ 0.50; large effect ≥ 0.80) [33]. Differences were considered significant when *p* < 0.05. The sample size was calculated using the G*Power V.1.1.3 software (Dusseldorf, Germany). A target of 20 participants was set, as this was estimated to provide 85% power (2-tailed, *p* < 0.05) to detect a 10% decrease in the amount of time spent in sedentary activities. An allowance for 10% attrition was included in determining the required sample size.

## 3. Results

Table 1 presents the participant demographics. Twelve (66.7%) participants were office workers, while six (33.3%) were full-time students. At baseline, participants were observed to be both high in sedentary time (9.4 ± 1.1 h/day) and yet achieving the recommended daily levels of moderate- (0.75 ± 0.2 h/day) and vigorous-intensity physical activity (0.44 ± 0.2 h/day) for adults [34]. Participants wore the personal activity monitor for an average 16.4 ± 0.2 h/day. No differences were observed for monitor wear time at either baseline or at the end of the 4-week intervention.

**Table 1:** Demographics of the study population.

Descriptors	Total (n = 18)	Male (n = 12)	Female (n = 6)	
Age (years)	29.0 ± 4.4	28.7 ± 4.9		29.5 ± 3.5
Height (cm)	171.9 ± 9.8	174.1 ± 10.8		167.6 ± 6.1
Weight (kg)	78.3 ± 20.6	84.6 ± 20.7		65.6 ± 14.5
BMI (kg/m^2^)	27.0 ± 6.5	28.0 ± 7.6		24.9 ± 2.5

Values are means ± 1 SD.

### 3.1. Efficacy of the Intervention

**Figure 1:**
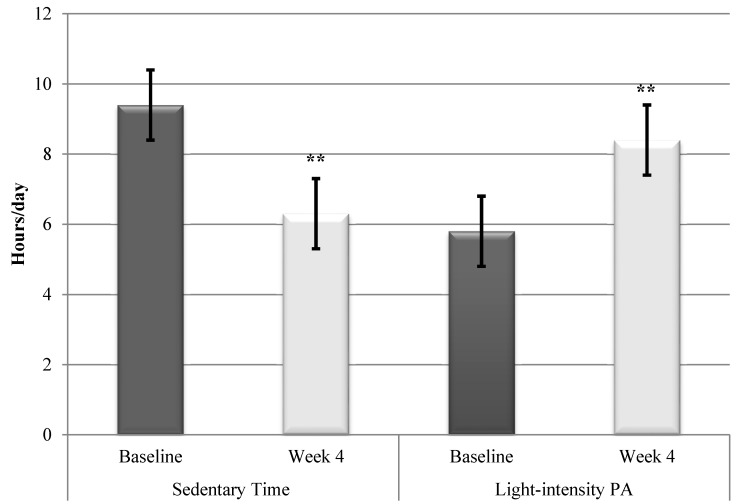
Decrease in sedentary time and increase in light intensity physical activity from baseline to week 4 (data from Gruve monitor). ** *p* < 0.001.

Analysis of the amount of time spent in sedentary activities revealed a significant decrease over the intervention period compared to baseline (*t* (17) = 9.5, *p* < 0.001) (Figure 1). A significant difference (*p* < 0.01) was found between the amount of time spent in daily sedentary activities measured at baseline (9.4 ± 1.1 h/day) and at the end of the 4-week intervention (6.3 ± 0.8 h/day) and the effect size was large (*d* = 3.0). This amounts to a decrease of 33% (3.1 h/day) in sedentary activities. Figure 1 also illustrates that a significant difference (*t* (17) = −7.7, *p* < 0.001) was found between the amount of time spent in light-intensity physical activity at baseline (5.8 ± 1.3 h/day) and at the end of the 4-week intervention (8.4 ± 1.0 h/day) and the effect size was also large (*d* = 2.2). This amounts to a daily average increase of approximately 45% (2.6 h/day) in light intensity physical activity.

**Figure 2:**
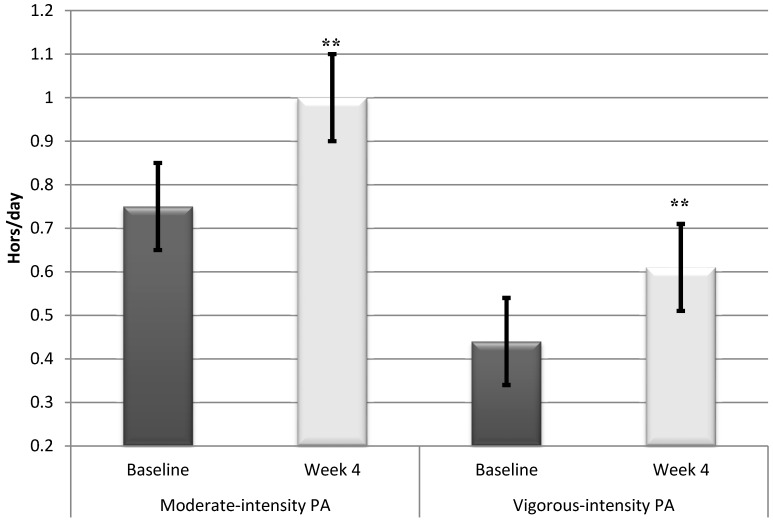
Increased time in both moderate and vigorous intensity physical activity from baseline to week 4 (data from Gruve monitor). ** *p* < 0.001.

A significant difference (*t* (17) = −4.8, *p* < 0.001) with a large effect size (*d* = 0.8) was found for the amount of time spent on daily moderate-intensity physical activity from baseline (0.75 ± 0.2 h/day) to the end of the 4-week intervention (1.0 ± 0.3 h/day). This difference amounts to an increase of 33% (0.25 h/day). Additionally, there was a statistically significant difference (*t* (17) = −3.0, *p* < 0.001) in the amount of time spent on vigorous-intensity physical activity from baseline (0.44 ± 0.2 h/day) to the end of the 4-week intervention (0.61 ± 0.3 h/day) and the effect size was medium (*d* = 0.5). This difference amounts to an average daily increase of approximately 38% (0.16 h/day) in vigorous-intensity physical activity (Figure 2). For energy expenditure, a statistically significant difference (*t* (17) = −7.0, *p* < 0.001), representing an increase from baseline to the end of the 4-week intervention, was detected. Finally, a significant difference with a large effect size (*d* = 0.9) was found in RMR between baseline (456.6 ± 176.2 kcal/day) and the end of the 4-week intervention (672.3 ± 274.3 kcal/day). This amounts to a daily increase above the RMR of 47% (216.7 kcal/day).

### 3.2. Self-Report Outcome (7-Day SLIPA Log)

Statistically significant differences in the mean amount of time spent in sedentary activities between the mean baseline to the end of the 4-week intervention were observed across the domains of *work* (*t* (17) = 3.5, *p* < 0.001, *d* = 0.4) (5.2 ± 1 h/day decreasing to 4.6 ± 1 h/day); *transportation* (*t* (17) = 3.9, *p* < 0.001, *d* = 1.0) (0.79 ± 0.4 h/day decreasing to 0.42 ± 0.2 h/day); and *leisure time* (*t* (17) = 5.9, *p* < 0.001, *d* = 1.5) (5.0 ± 0.9 h/day decreasing to 3.6 ± 0.9 h/day).

**Figure 3:**
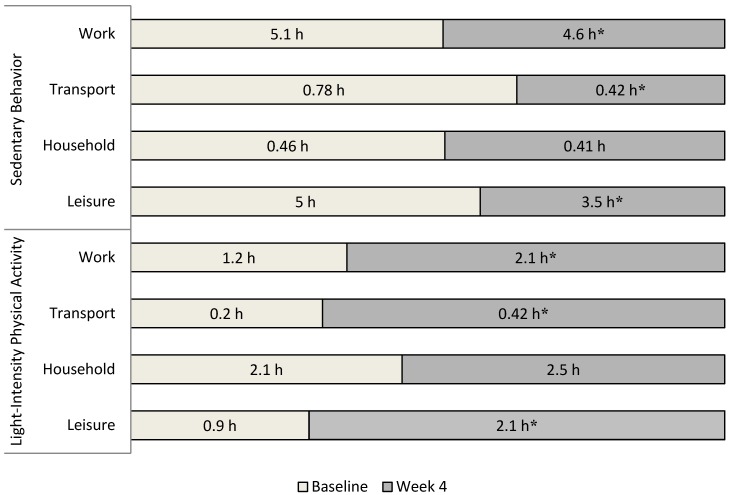
Time differences (h/day) spent in sedentary behavior and light-intensity physical activity across four daily life domains: work, transportation, household activities, and leisure time (based on the 7-day SLIPA Log). * *p* <0.01.

Significant differences between baseline and the end of the 4-week intervention were found in the amount of time spent in light-intensity physical activity across the domains of *work* (*t* (17) = −4.7, *p* < 0.001, *d* = 0.9) (1.2 ± 0.7 h/day increasing to 2.1 ± 1 h/day); *transportation* (*t* (17) = −11.8, *p* < 0.001 *d* = 2.2) (0.20 ± 0.1 h/day increasing to 0.42 ± 0.1 h/day); and *leisure time* (*t* (17) = −5.1, *p* < 0.001, *d* = 3.2) (0.9 ± 0.7 h/day increasing to 2.1 ± 1 h/day). There were no significant differences between baseline and the end of the 4-week intervention for sedentary activities (*t* (17) = −1.6, *p* = 0.116) (2.1 ± 2 h/day increasing to 2.5 ± 2 h/day) or light-intensity physical activity (*t* (17) = 1.2, *p* = 0.215) (0.46 ± 0.2 h/day decreasing to 0.41 ± 1 h/day) in the *household* activities domain (Figure 3).

## 4. Discussion

An increasing body of evidence suggests that multi-component interventions appear to be more effective for changing behavior than single-component efforts [35,36]. The current study involved a multi-dimensional behavioral intervention using an online personal activity monitor with a built-in vibrating function that indicated to participants when they had been sedentary for longer than the threshold. This reminder to stand up and move around provided a helpful prompt for behavior change. The online software enabled participants to visualize their daily activity patterns with simple 24 h/day graphs and charts, enabling them to self-monitor physical activity levels and energy expenditure and to achieve the set goals. Consequently, a significant reduction in the amount of time spent in sedentary activities was observed.

Currently, there are few intervention studies dedicated to reducing sedentary behavior that use objective methods for quantifying the changes induced. To some extent, the results from this study are consistent with recent research, although differences exist regarding sample demographics, intervention objectives and measurement instruments. For example, one study examined the efficacy of 4-week intervention by breaking up prolonged periods of sedentary behavior time with brief physical activity breaks (e.g., walking). Thirty overweight and obese adults were regularly prompted via an Android smartphone (*i.e.*, Samsung Exhibit 4G SGH-T759) [22]. The researchers found that participants using the smartphone-based intervention reduced their sedentary time by 2 h/day from the average 9.8 h/day shown at baseline (approximately 14.8%). Another study involving overweight and obese office workers examined the feasibility of reducing the amount of time spent in sedentary activities by using targeted messages. These targeted messages contained information about potential health risks associated with sedentary behaviors and recommended they replace time spent in sedentary activities with standing and light-intensity activity [37]. Time spent in sedentary activities was measured using wearable monitors and self-reporting tools. The findings showed that participants reduced the amount of time they spent in sedentary activities by 48 min/day over a 16-h waking day [37]. Adams and colleagues [38] recently reported success with an intervention that aimed to reduce sedentary behavior among obese women. The researchers incorporated face-to-face sessions, email messages and pedometer information for informed self-evaluation and goal setting. They reported that participants were able to decrease sedentary behavior time and increase physical activity levels.

Participants in the current study replaced the time spent in sedentary activities by significantly increasing light and moderate physical activity. Unexpectedly, our investigation found a significant increase in time spent in vigorous physical activity between baseline and the end of the 4-week intervention (*p* < 0.001). The medium effect size of this pre/post difference in a sedentary adult sample is surprising, given that the personal activity monitor was developed to promote daily non-exercise activity thermogenesis (NEAT) activities, which are composed mainly of energy expenditures related to daily physical activity of light-to-moderate intensity [39].

While the amount of time spent in sedentary activities decreased and light-intensity physical activity increased, it is important to consider pre-post changes that occurred across the specific SLIPA-determined daily life domains (work, transportation, household activities and leisure time), as these are likely to differ in terms of control over behavioral change [30,40]. The 7-day SLIPA Log data analysis revealed that there were three domains (work, transportation and leisure time) in which participants reduced the amount of time they spent in sedentary activities and increased the time they spent in light physical activity between baseline and the end of the 4-week intervention (see Figure 3). Large effect sizes were associated with a reduction in the amount of time spent in sedentary activities and increased light-intensity physical activities within the leisure time domain. One possible explanation for this result may be due to the questions utilized within the leisure time domain. These questions primarily reference activities that generally occur in mid-to-late evening (from dinner until bedtime). Interestingly, the mid-evening period, according to the Gruve data, was an important time for attaining the daily energy expenditure goals. For most of the participants, this is the epoch when the daily activity halo bar color frequently lay between the orange and blue LED. In an attempt to achieve their daily monitor Green Goal during this mid-evening leisure time, many participants appeared to replace the time they spent in sedentary activities (e.g., sitting, watching TV) with standing or walking (perhaps while still watching TV). This low intensity activity behavior contrasts with results from previous research, where sitting during leisure time (*i.e.*, sitting, watching TV) is the most frequent evening activity among adults [41,42].

### Limitations and Strengths

The main limitation of the study is the limited generalizability due to a small sample size that was primarily comprised of younger adult participants. Although the Gruve monitor has been validated in laboratory conditions for sitting, standing and walking, it has not been fully validated in “free living” conditions. A further limitation will be the threat to the internal validity which pre-test post-test designs invoke. Such a design is helpful, however, with future hypothesis generation to assist in determining whether a more rigorous study design is warranted. Participants were asked to charge the monitor battery during sleep. The monitor battery needed to be recharged every 2–3 days, and a full charge normally took between 2 and 4 h. Therefore, if the participants were to charge the battery during waking time, this event could lead to an unintentional, yet additional, 2–4 h in the amount of time spent in sedentary activities being recorded. This study has several strengths. It is the first intervention study which has reported such a large and significant decrease in sedentary activities among sedentary adults during free-living activities using the Gruve monitor as an intervention tool. As part of the inclusion criteria, the participants’ sedentary behavior was determined via the self-report (using the IPAQ), thus they were engaged in high volumes of sedentary behavior before participating in this study. Consequently, we were confident that our participants met the most recent definitions of sedentary behaviors [6]. In addition, the study employed the 7-day SLIPA Log self-report measures to identify sedentary behavior across all daily life domains. Future studies should include a larger sample size, a longer time period with a more extensive age range, randomized controlled trials. It would also be prudent to examine the health benefits of decreases in the amount of time spent in sedentary activities in a longitudinal study.

## 5. Conclusions

This lifestyle study involving sedentary adults suggests that, when engaging with the online personal activity monitor, individuals decreased (by 33%) the amount of time they spent in sedentary activities. No previously published research has found such a large reduction in the amount of time spent in sedentary activities. Furthermore, participants increased their daily light- and moderate-intensity physical activity and energy expenditure. In addition, due to the time stamp on the Gruve output, it was evident the increased physical activity and reduction in sedentary time were most likely to occur within the daily leisure time domain. Future investigations should focus on determining whether this intervention has a sustainable impact in sedentary behavior and related health outcomes.
